# Toward the Integration of an Attract-and-Kill Approach with Entomopathogenic Nematodes to Control Multiple Life Stages of Plum Curculio (Coleoptera: Curculionidae)

**DOI:** 10.3390/insects11060375

**Published:** 2020-06-17

**Authors:** Jaime C. Piñero, David Shapiro-Ilan, Daniel R. Cooley, Arthur F. Tuttle, Alan Eaton, Patrick Drohan, Kathleen Leahy, Aijun Zhang, Torri Hancock, Anna K. Wallingford, Tracy C. Leskey

**Affiliations:** 1 Stockbridge School of Agriculture, University of Massachusetts, Amherst, MA 01003, USA; dcooley@umass.edu (D.R.C.); aftuttle3@gmail.com (A.F.T.); 2 USDA ARS Southeastern Fruit and Tree Nut Research Laboratory, Byron, GA 31008, USA; david.shapiro@usda.gov; 3 University of New Hampshire Cooperative Extension, Durham, NH 03824, USA; Alan.Eaton@unh.edu (A.E.); Anna.Wallingford@unh.edu (A.K.W.); 4 Department of Ecosystem Science and Management, Penn State University, University Park, PA 16802, USA; pjd7@psu.edu; 5 Polaris Orchard Integrated Pest Management for New England, Colrain, MA 01340, USA; polaris2@rcn.com; 6 USDA ARS Invasive Insect Biocontrol and Behavior Laboratory, Beltsville, MD 20705, USA; aijun.zhang@usda.gov; 7 USAMIRIID, Fort Detrick, Frederick, MD 21702, USA; torritj@hotmail.com; 8 USDA-ARS, Appalachian Fruit Research Station, Kearneysville, WV 25430, USA; tracy.leskey@usda.gov

**Keywords:** aggregation, semiochemicals, integrated pest management, biological control, behavior

## Abstract

Efforts to reduce insecticide inputs against plum curculio, *Conotrachelus nenuphar*, a key pest of apples in eastern North America, include perimeter-row insecticide sprays applied after the whole-orchard petal fall spray to manage dispersing adults and, more recently, insecticide sprays confined to odor-baited trap trees. Entomopathogenic nematodes (EPNs) are virulent to ground-dwelling stages of *C. nenuphar*, and may be applied to the ground underneath trap-tree canopies. Here, we (1) compared the efficacy of the odor-baited trap tree approach with grower-prescribed (=grower standard) sprays to manage *C. nenuphar* populations over a six-year period in seven commercial apple orchards in New England; and (2) assessed the performance of the EPN *Steinernema* *riobrave* at suppressing ground-dwelling stages of *C. nenuphar*. In addition, the performance of *S. riobrave* was compared against that of *S.* *carpocapsae* and *S. feltiae* in one year. Across the six years, percent fruit injury on trap tree plots averaged 11.3% on odor-baited trap trees and 1.4% on unbaited trees in grower standard plots, highlighting the ability of trap trees to aggregate *C. nenuphar* activity and subsequent injury. Mean percentage injury on fruit sampled from interior trees, the strongest measure of treatment performance, in trap tree plots did not differ significantly from that recorded on interior trees in grower standard spray plots (0.95 vs. 0.68%, respectively). *Steinernema riobrave* consistently reduced *C. nenuphar* populations as indicated by the significantly lower number of adult *C. nenuphar* that emerged from the soil, when compared to water control. *Steinernema* *carpocapsae* and *S. riobrave* performed similarly well, and both EPN species outperformed *S. feltiae*. Our combined findings indicate that an IPM approach that targets multiple life stages of *C. nenuphar* has the potential to manage this pest more sustainably in a reduced-spray environment.

## 1. Introduction

The plum curculio, *Conotrachelus nenuphar* (Herbst.) (Coleoptera: Curculionidae), stands as one of the most devastating and persistent native pests of apple in eastern North America [1,2]. Damage to fruit by *C. nenuphar* is initiated as soon as fruit reach a diameter of 6–7 mm, and results from feeding and oviposition scars produced by adult females and from burrows made by the larvae [3]. Currently, to provide commercially acceptable levels of *C. nenuphar* control, many fruit growers in the northeast continue to apply up to three whole-orchard broad-spectrum insecticide sprays [4,5,6]. This situation makes *C. nenuphar* a major obstacle to ecological and sustainable pest management programs in tree fruit orchards [2,7,8].

For many apple growers in MA and other New England states, ≤1% is an economically acceptable level of whole-block fruit injury by *C. nenuphar* [6,8]. To successfully manage *C. nenuphar* in a reduced-spray environment that would result in tolerable injury levels, then it is imperative that alternative management strategies consider the ecology and behavior of the target pest, as well as biological control options that may be available. Approaches that have received attention for controlling *C. nenuphar* in ways that could result in reductions in the overall amount of insecticide applied against this pest include: (1) an attract-and-kill system, involving the use of attractive lures and targeted sprays against adults [8]; and (2) biological control through the application of entomopathogenic nematodes (EPNs), targeting larvae in the soil [9,10,11,12].

A novel attract-and-kill strategy, involving odor-baited trap trees for direct control of *C. nenuphar*, was developed by Leskey et al. [8], based on a seminal work by Prokopy et al. [13,14]. This attract-and-kill approach calls for baiting the branches of several perimeter-row trees with a synergistic two-component lure [15,16] comprised of the plant volatile benzaldehyde (BEN), in association with grandisoic acid (GA), the synthetic *C. nenuphar* aggregation pheromone [17]. Odor-baited trap trees aggregate adult *C. nenuphar*, and the canopies of such trees are subsequently sprayed with insecticides, while the other trees in the orchard block remain pesticide-free. Restricting post-petal fall treatments to a few perimeter-row trees, rather than the perimeter rows or the entire orchard, can reduce insecticide treatment by more than 90% [8].

Entomopathogenic nematodes are commercially available biocontrol agents used to control a wide variety of economically important insect pests in orchards and other cropping systems [18]. While the use of entomopathogenic nematodes (EPNs) for biological control of *C. nenuphar* is not a new concept, in recent years, it has received renewed attention, in light of its potential integration with attract-and-kill systems. The results from previous studies, e.g., [10,11,12] indicate that *Steinernema riobrave* Cabanillas, Poinar and Raulston is one of the most virulent EPN species to the ground-dwelling stages of *C. nenuphar*. For example, relative to the untreated check, S. *riobrave* caused 85.0% and 97.3% control in 2011 and 2012, respectively, in Belchertown, Massachusetts, and 100% control in West Virginia on both years. Another nematode species, *S. feltiae* (Filipjev), caused 0% and 84.6% suppression in 2011 and 2012, respectively, in Belchertown, and 78.2% and 69.7% suppression in West Virginia [12].

The main goal of this study was to explore, in multiple commercial apple orchards and over a six-year period, the efficacy of the trap tree approach as an attract-and-kill strategy against *C. nenuphar*. Parallel investigations on the effectiveness of EPNs applied to the soil to target immature stages of *C. nenuphar* were conducted. In particular, we sought to address the following questions: (1) does the presence of the BEN+GA lure in trap trees consistently result in significant aggregation of fruit injury within these tree canopies compared to unbaited tree canopies? (2) can the orchard-wide injury by *C. nenuphar* be maintained at economically acceptable levels under a reduced spray scenario involving the trap tree management strategy? (3) does the level of injury received by odor-baited trap trees extend to neighboring trees? and (4) are EPNs applied to the soil underneath trap trees effective at suppressing *C. nenuphar* in multiple orchards over multiple years? Our overall goal was to integrate the use of odor-baited trap trees with EPNs, as an integrated pest management (IPM) program that targets multiple life stages of *C. nenuphar.*

## 2. Materials and Methods

### 2.1. Study Sites

This investigation was conducted over a 6-year period in commercial orchards located in Massachusetts (Clark Brothers Orchards in Ashfield; Clarkdale Fruit Farms in Deerfield; University of Massachusetts Cold Spring Orchard in Belchertown), New Hampshire (Apple Hill Farm in Concord; Gould Hill Farm in Contoocook; Poverty Lane Orchards in Lebanon), and Vermont (Scott Farm in Dummerston). Not every orchard participated in this study on each year. Table 1 lists the participant orchards.

### 2.2. Study 1: Attract-and-Kill of C. Nenuphar Using Odor-Baited Trap Trees

For each participant orchard, we evaluated two treatments: (1) odor-baited trap tree management strategy; and (2) grower standard plots that received insecticide treatment as prescribed by the grower. Within each orchard, two experimental plots were established. One plot was randomly assigned to the trap tree treatment, and the second plot was selected for grower standard sprays. The average area of experimental plots was 1.44 and 1.13 ha for the trap tree and the grower standard plots, respectively. The same two plots within an orchard were used on each year, but the assignation of trap tree and grower standard treatments was switched on most years. All orchard plots received a full-block spray of insecticide (most commonly an organophosphate, see below) by the time of petal fall. In New England, this is a standard practice targeting *C. nenuphar* and other pests (e.g., oriental fruit moth, *Grapholita molesta* [Busck] [Lepidoptera: Tortricidae]) that may have penetrated into interior trees [19,20]. Subsequent sprays were applied to either trap trees only in trap tree plots, or as prescribed by the growers in the grower standard plots. Across the six years, the post-petal fall grower standard approach involved 1–2 full block sprays (60% of the orchards) or 1–2 perimeter-row insecticide sprays (40% of the orchards). The post-petal fall sprays (1–2 applications) targeting *C. nenuphar,* the most important early-season pest, were applied starting 7–10 days after the petal fall spray. Insecticide sprays against another key pest, *Rhagoletis pomonella* (Walsh) (Diptera: Tephritidae), commence later in the summer.

*Trap tree plots.* During full bloom on each year, selected perimeter-row trap trees were baited with four BEN dispensers and one GA dispenser (ChemTica, San Jose, Costa Rica). Each BEN dispenser was suspended inside of an inverted colored plastic drinking cup (volume = 266 mL) (Solo Cup Co., Urbana, IL, USA), to minimize the potential negative impact of ultraviolet light on the stability of BEN [16]. The GA dispensers contained 35 mg of grandisoic acid, according to the manufacturer, and they were expected to release ~0.14 mg/day per trap tree at 25 °C [21]. The four BEN dispensers were deployed equidistantly throughout the outer third of the canopy, whereas the GA dispenser was deployed near the center of the tree [8,13,14]. All BEN lures were left in place for the entire period of *C. nenuphar* activity, while the GA lures were replaced once, typically 4 weeks after initial deployment. The distance between trap trees was 35 m, based on Prokopy et al. [14] who reported that the distance over which an odor-baited trap tree was effective in aggregating damage to fruit extended to at least 33 m. On average, there were eight trap trees per ha.

*Pesticides used by growers.* Grower cooperators most commonly applied the organophosphate phosmet (Imidan 70-W^®®^; Gowan Co.), the neonicotinoid thiacloprid (Calypso^®®^, Bayer CropScience LP), the oxadiazine indoxacarb (Avaunt^®®^, FMC) or the pyrethroid esfenvalerate (Asana XL^®®^, Valent) at recommended rates, against *C. nenuphar*. Fungicides to control scab and other summer diseases were applied as deemed necessary by the growers.

*Data collection*. Treatment performance was assessed for each orchard by means of fruit injury evaluations conducted between 23 Jun and 5 July of each year. The total number of fruit with oviposition scars was recorded, based on a sample of 25 fruit/tree from trap trees in the trap tree plot and from unbaited (control) trap trees in the grower standard plot. To quantify the level of spillover to trees immediately adjacent to the odor-baited trap tree, 25 fruit per tree were sampled from six peripheral trees (three to the right and three to the left) next to the trap tree and the control trap tree (in the grower standard plot). Additionally, we sampled 25 fruit from each of three trees behind each odor-baited trap tree and each control trap tree [8]. To provide a measure of the efficacy of each treatment regime to protect interior-plot fruit from *C. nenuphar* damage 20 interior trees (25 fruit/tree) were sampled within each plot. The mean number (±SD) of fruit sampled across years was 8622 (±4500) and 6890 (±3710) for the trap tree plots and for the grower standard plot, respectively. In all, 92,676 fruit were sampled across all years and orchards.

### 2.3. Study 2: EPN Application against Ground-Dwelling Stages of C. Nenuphar

Here, we evaluated the efficacy of EPN application formulated in water targeting immature stages of *C. nenuphar* in the soil. The treatments were compared against a water-only control. Two experiments (described below) were conducted.

**Figure 1:**
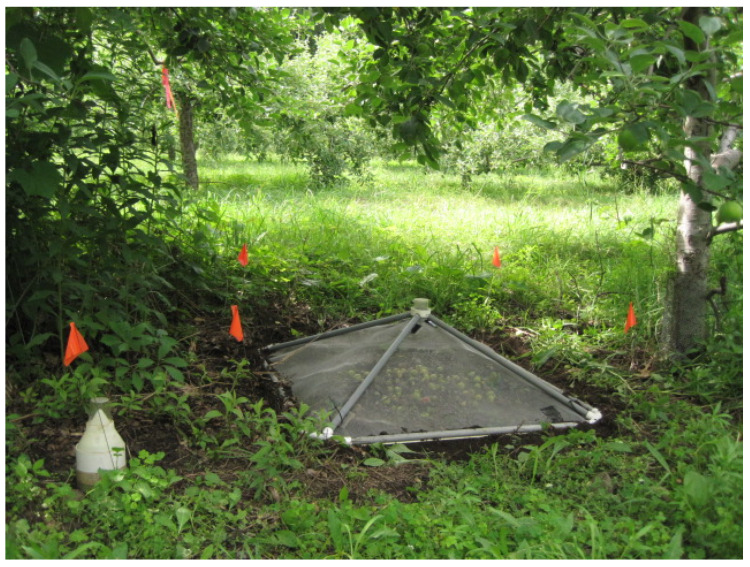
Depiction of the polyvinyl chloride (PVC) enclosure (**left**) and pyramidal emergence cage (1 m × 1 m at the base) (**center**) used for the evaluation of entomopathogenic nematodes in the second study.

*Trap types.* We used two approaches to measure the number of adult *C. nenuphar* emerging from the soil after EPN application. The first approach involved mini-plot cylindrical enclosures (Figure 1) made of polyvinyl chloride (PVC) (11.43 cm in diameter and 17 cm in height) (hereafter referred as to PVC enclosures), which were buried to 15 cm deep [12]. After EPN application (see below), a boll weevil trap, consisting of a green plastic cylindrical base, a molded screen cone and a collection chamber, was buried using each enclosure as a ‘sleeve’. As they emerged, adult *C. nenuphar* were collected in the collection chamber. This type of experimental arena was used in 2013, 2014, and 2015.

The second approach consisted of pyramidal emergence cages (1 × 1 m at the base) made of PVC and steel screen [22] (Figure 1). A plastic conical device that topped each cage permitted the capture of adult *C. nenuphar* that, upon adult emergence, walked upward on the interior surface of the capturing device. One pyramidal emergence cage was placed underneath the canopy of each trap tree (the same tree used for the PVC enclosure). Emergence cages were used in 2013–2015, 2018, and 2019.

*Experiment 1*. This experiment was undertaken in 2013, 2014, 2015, and 2018 in trap tree plots. On each year, about 36 trap trees (6 per orchard) were selected for the application of either, *S. riobrave* (rate: 100 IJs/cm^2^) or water (control). The application of EPNs or water was done using 3.78 lL plastic containers, and treatments were assigned at random. For the PVC enclosures, 30 fully-developed *C. nenuphar* larvae were placed inside the enclosures 24 h prior to EPN application. For emergence pyramidal cages, approximately 75 *C. nenuphar*-infested fruit were placed on the center of each caged area, 24 h before EPNs were applied, to allow the larvae to crawl in soil. Except for natural precipitation, no additional water was added to the experimental units after EPN application.

*Experiment 2.* The 2019 experiment aimed to compare the effectiveness of commercial formulations of *S. riobrave*, *S. carpocapsae* (Weiser), and *S. feltiae* against a water control, using pyramidal emergence cages. The virulence of the three EPN species evaluated was quantified in a preliminary test under laboratory conditions using greater wax moth, *Galleria mellonella*, (L.) (Lepidoptera: Pyralidae) larvae, a highly susceptible host. The moth larvae were purchased from Bestbait (Lakeside Marblehead, OH, USA). Ten *G. mellonella* larvae were placed on Petri dishes (10 cm diameter), loaded with 1.5 cm of moist sand, and 20 mL of each of the three EPN species (rate: 100 IJ/cm^2^) or the water control was applied to different Petri dishes. Each treatment was replicated 4 times. All three EPN species caused 100% mortality of *G. mellonella* larvae within 24 h, confirming the strong activity of the commercial formulations of EPNs used for the field study.

The field experiment took place at the University of Massachusetts Cold Spring Orchard (Belchertown, MA, USA) from 24 July to 30 August, 2019. In early July 2019, we collected infested apple fruitlets presumably infested with *C. nenuphar* from unmanaged trees in the Belchertown area. Upon collection, the fruit was mixed and stored at ambient temperature for about 10 days, to allow *C. nenuphar* larvae to continue developing. On 24 July, the fruit was transported to an unsprayed section of the orchard. Seventy-five fruit were placed underneath the canopies of each of 20 apple trees, within a 1 m^2^ area. EPNs were applied at a rate of 200 IJ/cm^2^ using 3.78 L of water, and the same amount of water alone was applied to the control. After treatment application, the emergence cages were placed on the ground, covering the fruit, and the edges of the cages were buried in the soil to ensure the emerged adults would not escape. Pieces of apple were placed inside the emergence cone as an attractant as soon as the first adult *C. nenuphar* was captured in the topping device. Each of the treatments (three nematode species) and the control were replicated five times. No additional water (except for natural precipitation) was added to the cages.

*Source of EPNs and application dates.* For the 2013–2015 studies, the EPN *S. riobrave* (355 strain) was shipped overnight from the USDA ARS Southeastern Fruit and Tree Nut Research Laboratory in Byron, GA, USA, to the field locations. For the 2018 evaluation, *S. riobrave* (355 strain) formulated as Nemasys^®®^ R was provided by BASF (Research Triangle, NC, USA). EPNs were applied on 7 July, 8, July, 14 July, and 16 July, for 2013, 2014, 2015, and 2018, respectively. All EPNs used in 2019 were purchased from Arbico Organics (Tucson, AZ, USA; https://www.arbico-organics.com), given that our goal was to evaluate EPN formulations (NemaSeek Sr™, NemaSeek Sf™, NemaSeek Sc™) that are commercially available to apple growers. The presumed strains were Sr ‘355′ strain, Sc ‘all’ strain, and sf ‘SN’ strain. Upon arrival, the nematodes were stored under refrigeration until applied to field sites. In 2019, EPNs were evaluated on 24 July.

*Data collection.* Two weeks after EPN application, the number of adult *C. nenuphar* collected in the experimental arenas (PVC enclosures and emergence cages) was recorded on a weekly basis for four weeks (experiment 1), or twice a week for four weeks (experiment 2). All insects were removed from the capturing devices.

### 2.4. Weather

Ambient temperature and precipitation data were obtained from on-site weather stations and from the Cornell University Network for Environment and Weather Applications (NEWA) (Geneva, NY, USA; http://newa.cornell.edu). Soil moisture and temperature data were collected using a Decagon 5TE soil moisture and temperature sensor array and Em50 logger. Prior to site installation, all sensors were calibrated, per Decagon recommendations [23], to a silt loam texture to match study location surface soil texture. Four locations were chosen within the perimeter of monitored trees in an orchard. At each sampling tree, the 5TE sensor was installed at 5 cm depth, and data was recorded at 10 min intervals during the growing season (growing season varied by year) on the Em50 logger. Soil data from the UMass Cold Spring Orchard were recorded using HOBOlink^®®^.

### 2.5. Characterization of Soil Types

Soils monitored in this study were mapped by the USDA-NRCS and mapping data for each farm was confirmed in the field. Soil information stems from the USDA Official Series Descriptions [24], and is presented in detail in Appendix A.

### 2.6. Statistical Analyses

For the first study, we calculated, for each sampled tree, the proportion of fruit that had *C. nenuphar* oviposition injury out of 25 sampled fruit. Data were converted to arc-sin prior to analyses. Two preliminary analyses were conducted. The first one involved *t*-tests, to ascertain the effect of insecticide spray type (perimeter-row sprays versus full block sprays) on the level of *C. nenuphar* injury to fruit sampled from interior trees. No significant differences were detected (*p* > 0.05) across spray types, so the connotation of ‘grower standard’ was valid. The second preliminary analysis involved generalized linear mixed models assuming a Poisson distribution, which compared the effects of ‘treatment’ (trap tree versus grower standard) (fixed effect) and ‘orchard’ and ‘year’ (both used as random factors), and the 2- and 3-way interactions among them. Overdispersion was tested by looking at deviance goodness of fit test using a log link function. The interaction of ‘treatment’ and ‘orchard’ was significant (df_5,15_ = 4.3; *p* = 0.012). Bonferroni-corrected *t*-tests were used to compare, for each year, the proportion of fruit injured by *C. nenuphar* in: (1) odor-baited trap trees in trap tree plots versus unbaited control trees in grower standard plots; and (2) average interior injury recorded in trap tree plots versus grower standard plots. The latter comparison provided the strongest measure of the effectiveness of the two management strategies evaluated here to protect fruit from *C. nenuphar*.

One-way analyses of variance (ANOVA) were conducted separately for trap tree plots and for grower standard plots to compare: (1) the average injury on trap trees versus the average injury on lateral perimeter trees (combining trees on the immediate left and right of trap trees); and (2) the average injury on trap trees versus the average injury recorded on the three trees located behind the trap tree.

For the EPN evaluations, the cumulative number of *C. nenuphar* adults that emerged from PVC enclosures and from emergence cages was compared statistically among treatments, using non-parametric Wilcoxon Matched Pairs Tests (EPN versus water control; 2013–2015 and 2018 data) and Kruskal-Wallis (three EPN treatments versus a water control, 2019 data). All analyses were conducted using STATISTICA v.13 (TIBCO Software Inc., Palo Alto, CA, USA).

## 3. Results

### 3.1. Study 1: Attract-and-Kill of C. Nenuphar Using Odor-Baited Trap Trees

**Figure 2:**
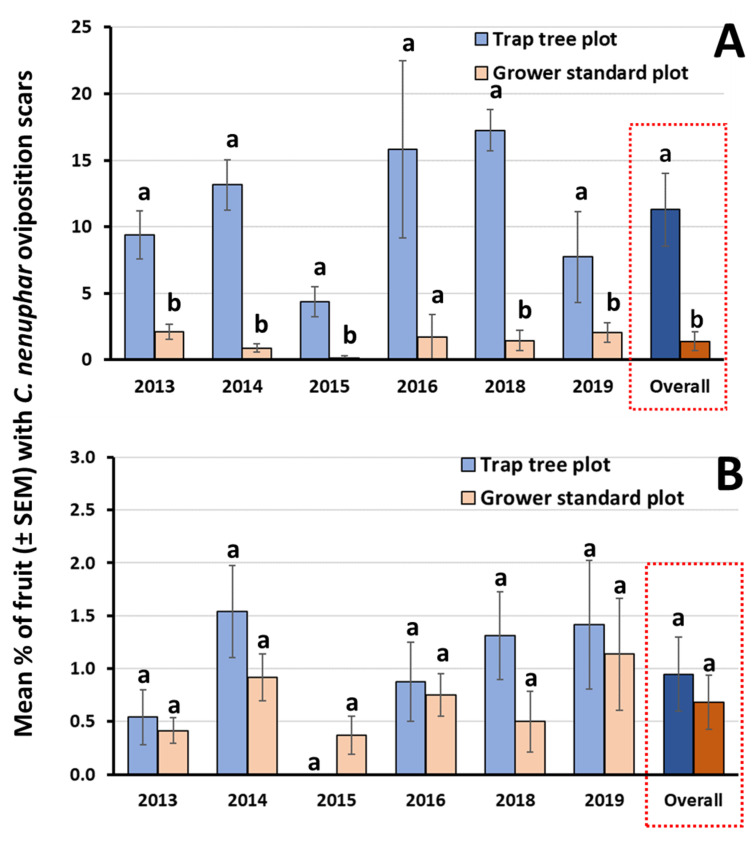
For each year and across all six years, level of fruit injury caused by *C. nenuphar* to (**A**) trap trees in trap tree plots and control (unbaited) trees in grower standard plots, and (**B**) interior trees, the strongest measure of treatment performance, comparing the two treatment regimes. Means within a panel and for each pair of bars capped with different letters are significantly different (*t*-tests: *p* < 0.05).

For the first question (“does the presence of the BEN+GA lure in trap trees consistently result in significant aggregation of fruit injury in these specific tree canopies compared with unbaited tree canopies?”), we found that the level of fruit injured by *C. nenuphar* within the canopies of odor-baited trap trees ranged from 4.4% (in 2015) to 17.3% (in 2018) in trap tree plots. In contrast, in grower standard plots the level of *C. nenuphar* injury on control (unbaited) trap trees ranged from 0.2% (in 2015) to 2.1% (in 2013). Significant differences in the mean percent injury between odor-baited trap trees and unbaited control trees were recorded on each year (2013–2015, 2018–2019 data) (*t*-tests, df = 10; *p* < 0.05 in all cases). In 2016, differences were marginally significant (*p* = 0.058), due to a high variability among samples. Across all six years, mean percent fruit injury was significantly greater (*p* < 0.01) in trap trees (11.3%) than in control trees (1.4%) (Figure 2A).

The results generated to address question (2) (“can the orchard-wide injury by *C. nenuphar* be maintained at economically acceptable levels under a reduced spray scenario involving the trap tree management strategy?” provided a measure of the efficacy of each treatment regime to protect interior-plot fruit. For each year, and across all years, the mean percent injury in interior trees located in trap tree plots did not differ significantly from that recorded in plots subject to grower standard sprays (*t*-tests, df = 10; *p* > 0.05 for all 6 years) (Figure 2B).

**Figure 3:**
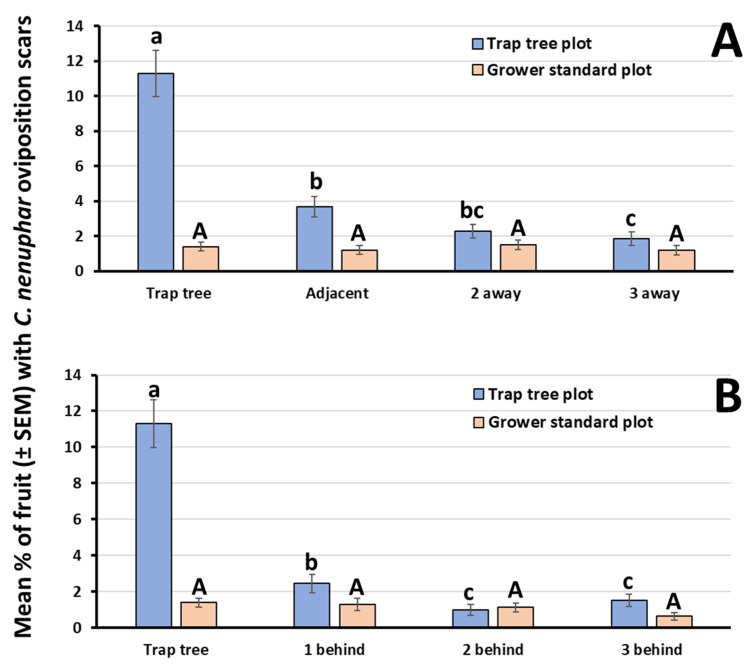
For the quantification of potential spillover effect, level of fruit injury caused by *C. nenuphar* to (**A**) odor-baited trap trees in trap tree plots, control (unbaited) trees in grower standard plots, and peripherally located neighboring trees; and (**B**) odor-baited trap trees in trap tree plots, control (unbaited) trees in grower standard plots, and to trees located behind (i.e., in interior rows). Within a panel and for each treatment, bars capped with different letters are significantly different (analysis of variance (ANOVA) *p* < 0.05).

For the third question (“does the level of injury spill over to neighboring trees?”), the two-way ANOVA revealed a significant interaction between treatment and location of perimeter-row trees neighboring the trap tree (F_3,182_ = 22.5, *p* < 0.001). Across all years and orchards, the average level of injury caused by *C. nenuphar* in odor-baited trap trees (11.3%) in trap tree plots was significantly greater than that recorded in any laterally located peripheral trees (3.7, 2.3 and 1.8%, for adjacent trees, and for trees located two away, and three away, respectively) (Figure 3A). In contrast, in grower standard plots the level of injury recorded in the control tree (1.4% on average) did not differ statistically from that recorded in the most adjacent perimeter-row trees (1.2%) or in trees located further away (1.5 and 1.2% for trees located two away and three away, respectively) (Figure 3A).

For the assessment of spillover effect to trees located behind the trap tree (i.e., going into the interior of the plot), we found that the interaction treatment*location was significant (F_3,172_ = 25.6, *p* < 0.001). In trap tree plots, significantly (about 4.6 times) less fruit injury occurred on the first tree behind (2.4%) the odor-baited trap tree, compared to the trap tree (11.3%) (Figure 3B). In contrast, no significant differences in level of fruit injury by *C. nenuphar* were noted for any of the trees sampled in grower standard plots (average injury ranged from 0.6 to 1.4%) (Figure 3B).

### 3.2. Study 2: EPN Application against Ground-Dwelling Stages of C. Nenuphar

The application of *S. riobrave* to the soil underneath trap trees consistently resulted in significant reductions in the number of summer-generation *C. nenuphar* that emerged from the soil, when compared to the water control (Table 2). In 2013, 2014, and 2015, significantly fewer adult *C. nenuphar* were recovered from PVC enclosures that received *S. riobrave* compared to the water control. For pyramidal emergence cages, significantly fewer adult *C. nenuphar* were recovered when *S. riobrave* was applied, when compared to the water control on each year.

Results from the 2019 field experiment revealed significant differences between treatments (Table 2). *Steinernema riobrave* and S. *carpocapsae* caused the greatest suppression of *C. nenuphar*, when compared to water control, whereas *S. feltiae* did not reduce *C. nenuphar* (Table 2).

## 4. Discussion

In the absence of semiochemicals, the current reduced-spray approach for *C. nenuphar* management practiced in some New England orchards calls for a whole-block application of insecticide shortly after petal fall, followed by one to three insecticide applications confined exclusively to peripheral-row trees [19,20]. The whole-orchard insecticide spray recommendation targets adult *C. nenuphar* that have either penetrated into orchard blocks by petal fall [3,20] or may have overwintered inside the blocks [22]. The pre-petal fall population likely represents the majority of the immigrating *C. nenuphar* population [25]. The New England recommendation to apply perimeter-row insecticide after the petal fall spray is based on the documented tendency of adult *C. nenuphar* to stay on perimeter-row trees [19,20,26].

The identification of an attractive lure for *C. nenuphar* by Piñero and Prokopy [16] led to the development of an effective monitoring tool for this pest by Prokopy et al. [13,14]. By establishing a few odor-baited trees on perimeter rows of apple orchards, apple growers can monitor *C. nenuphar* oviposition activity accurately and inexpensively [6]. The attract-and-kill strategy developed by Leskey et al. [8,27] represented a new reduced input strategy for managing *C. nenuphar* in apple orchards, based on the application of insecticides to a few perimeter-row odor-baited trap trees, rather than the entire perimeter row. A similar approach has been developed to manage *C. nenuphar* in blueberries [28].

The results from our first trap tree-related question supported our first hypothesis that the presence of the synthetic lure composed of BEN+GA in trap trees results in significant aggregation of fruit injury in those canopies, compared with unbaited trees in grower standard plots. Overall, 8.1 times more fruit injury by *C. nenuphar* was found within the canopies of trap trees (11.3% on average) in trap tree plots, compared with unbaited ‘control’ trees (1.4% on average) in grower standard plots. Our findings support previous results of Leskey et al. [8] who reported, over a two-year period, that the level of fruit injury by *C. nenuphar* was significantly greater in trap trees, compared with unbaited neighboring trees. In that study, the attract-and-kill strategy involving odor-baited trap trees resulted in a reduction of ~70% total trees being treated with insecticide, compared with perimeter row sprays, and 93% compared with the conventional approach involving full orchard sprays [8].

Our second question was whether injury by *C. nenuphar* would be maintained at economically acceptable levels under a reduced spray scenario, involving the trap tree management strategy that targets the adult stage. Our results from this multi-year, multi-orchard evaluation indicate that the average level of injury by *C. nenuphar* to fruit sampled from the plot interior was statistically similar in trap tree plots (0.95%), and in plots subject to grower standard sprays (0.68%). Such levels of injury occurring on interior trees are acceptable by growers in New England [6].

One concern that was expressed in previous investigations by our group was whether the level of injury to odor-baited trap trees spills over to neighboring trees (question 3). Previously, when evaluated in a year with a comparatively high *C. nenuphar* pressure, Leskey et al. [8] reported a spillover effect, in the sense that trees neighboring the trap trees received a level of injury to fruit that did not significantly differ from injury recorded on odor-baited trap trees. Such a spillover effect, however, did not extend beyond a couple of trees adjacent to trap trees. In the study by Leskey et al. [8], it is conceivable that such a spillover effect may have been due, in part, to rainy weather that prevented growers from timely applying insecticide in the experimental blocks. Here, the average level of fruit injury recorded in trap trees was significantly greater (at least three times) than that occurring in the most proximal trees (i.e., located left and right of trap trees). However, because the mean injury recorded on the most proximal trees was significantly greater than that recorded on the three-away tree, then a relatively small spillover effect was detected. A similar result was found for trees located behind the odor-baited trap tree.

Our results from the second study involving EPNs confirmed that *S. riobrave* is effective at killing immature stages of *C. nenuphar* in the soil. Our results were consistent across years and orchards (data not shown), indicating that EPNs perform well across a variety of soil types and environmental conditions (Table 2) of northeastern states. The EPN *S. riobrave* was selected for most of our evaluations based on documented virulence against *C. nenuphar* in previous studies [10,11,12]. It is interesting to note that the high level of EPN efficacy was achieved without additional irrigation to the field plots, which is unusual for EPN applications in general [17], but confirms a previous field study targeting *C. nenuphar* [12]. Apparently, as in the previous study, the levels of precipitation and water-holding capacity of the soils in this New England region were sufficient, such that irrigation was not necessary.

While *S. carpocapsae* was not included in the 2013–2015 and 2018 field studies, this EPN species showed good field performance in our 2019 evaluation. In prior studies conducted in the laboratory that involved multiple soil types and temperatures, most comparisons indicated *S. carpocapsae* to be as virulent as *S. riobrave* against *C. nenuphar* larvae or adults [9,11]. Both *S. carpocapsae* and *S. riobrave* were found to be more efficacious than *S. feltiae* in several lab or field studies [9,10,11,12]. Going forward, it seems that the two most effective EPN species, *S. riobrave* and *S. carpocapsae*, should be tested at a larger scale.

The overall goal of this research is to integrate the use of the attract-and-kill strategy targeting adult *C. nenuphar* with biological control involving the application of EPNs targeting the soil-dwelling stages of the pest. This novel strategy is expected to reduce insecticide use, while promoting the biologically based management of multiple life stages of *C. nenuphar*. By only spraying odor-baited trees, the total number of trees that receive insecticide treatment can be reduced by more than 90%, compared to full-block sprays [8]. As shown here, EPNs can be applied to the soil in areas underneath the canopies of odor-baited trap trees, areas that are expected to hold greater densities of *C. nenuphar* compared to any other trees in the orchard. EPN applications are compatible with organic production. The economic feasibility of using EPNs applied underneath the canopies of trap trees is very promising because, even if high rates of nematodes are applied, such applications would only need to be made to a small proportion of the acreage.

## 5. Conclusions

In conclusion, the present study indicated that, over multiple years and locations, (1) odor-baited trap trees consistently aggregated fruit injury by *C. nenuphar*; (2) insecticide sprays confined to trap trees only after the petal fall spray resulted in similar level of fruit injury in interior trees, compared to plots that received grower-prescribed sprays; (3) small, yet statistically significant, spillover effects were noted in trap tree plots involving the trees most proximal to odor-baited trap trees; and (4) *S. riobrave* and *S. carpocapsae* are effective at suppressing the immature stages of *C. nenuphar*. Overall, this study supports a multi-stage IPM program system that integrates the use of synergistic lures and insecticide applications to the canopies of baited trees, and the timely application of *S. riobrave* or *S. carpocapsae* in the areas underneath trap trees, to suppress the ground-dwelling stages of *C. nenuphar*.

## Figures and Tables

**Table 1:** Participant orchards and weather data according to year. Empty boxes indicate no orchard participation on that year. For each year, mean air temperature (in °C) is presented from 15 May (an approximation to petal fall date, reflecting the onset of oviposition activity by *C. nenuphar*) to 30 June (general date reflecting the cessation of egg-laying activity). Total precipitation (PCPN) is shown in centimeters. Soil volumetric water content and temperature comprise a 3-year average (2010, 2011, 2013) for each site. Soil temperature and volumetric water content (m^3^/m^3^) values are presented for the period of 1 July to 31 August, the period that generally corresponded to the entomopathogenic nematodes (EPN) activity in the soil.

Orchard/Year		2013	2014	2015	2016	2018	2019	3-Year Avg. Volumetric Water Content	3-Year Avg. Soil Temp
Apple Hill (NH)	Air temp PCPN	17.2 26.1	16.9 16.3	17.7 15.1	18.2 8.6	17.1 9.6	16.6 21.6	0.18	19.21
Clark Brothers (MA)	Air temp PCPN	16.9 46.0	16.9 3.3	17.1 28.5				0.25	19.48
Clarkdale (MA)	Air temp PCPN	18.1 35.8	18.1 20.8	17.9 23.1		18.4 14.2	18.0 13.2	0.19	20.44
Cold Spring Orchard (MA)	Air temp PCPN					18.1 13.7	17.8 11.7	-----	21.2
Gould Hill (NH)	Air temp PCPN	17.4 35.0	17.7 13.7	14.4 16.7				0.20	19.34
Poverty Lane Orchards (NH)	Air temp PCPN	15.9 24.4	16.3 19.4	16.0 22.8	18.2 6.2	17.0 9.7	16.4 21.6	0.21	18.34
Scott Farms (VT)	Air temp PCPN	16.8 30.7	17.3 18.8	17.1 16.8				0.19	19.40

**Table 2:** Results of the second field study involving applications of entomopathogenic nematodes to areas underneath odor-baited trap trees targeting the soil-dwelling stages of *C. nenuphar*, according to year. For each evaluation, the cumulative number of adult *C. nenuphar* that emerged from the soil and were captured in the trapping devices (PVC enclosures and emergence cages) was compared statistically among treatments. Data from 2013–2015 and 2018 were analyzed using Wilcoxon signed-rank tests, whereas data from 2019 were analyzed using Kruskal-Wallis. Mean ± SEM values are presented in all cases for ease of interpretation.

Year	Treatment	Trap Type	Mean Cumulative Emergence of Adult *C. Nenuphar* (±SEM)	Outcomes of Analyses
**2013**	*S. riobrave* (*n* = 18)	PVC enclosure	0.00 **a**	Wilcoxon Matched Pairs Test; z = 2.85, *p* = 0.004.
	Control (*n* = 18)	PVC enclosure	2.22 (±0.6) **b**	
	*S. riobrave* (*n* = 15)	Pyramid	1.06 (±0.6) **a**	Wilcoxon Matched Pairs Test; z = 3.47, *p* < 0.001.
	Control (*n* = 15)	Pyramid	12.87 (±2.6) **b**	
**2014**	*S. riobrave* (*n* = 18)	PVC enclosure	0.00 **a**	Wilcoxon Matched Pairs Test; z = 2.27, *p* = 0.020.
	Control (*n* = 18)	PVC enclosure	0.78 (±0.3) **b**	
	*S. riobrave* (*n* = 15)	Pyramid	15.60 (±4.5) **a**	Wilcoxon Matched Pairs Test; z = 2.40, *p* = 0.016.
	Control (*n* = 15)	Pyramid	28.60 (±5.9) **b**	
**2015**	*S. riobrave* (*n* = 18)	PVC enclosure	0.50 (±0.6) **a**	Wilcoxon Matched Pairs Test; z = 3.39, *p* < 0.001.
	Control (*n* = 18)	PVC enclosure	3.33 (±0.6) **b**	
	*S. riobrave* (*n* = 16)	Pyramid	0.68 (±0.2) **a**	Wilcoxon Matched Pairs Test; z = 2.02, *p* = 0.043.
	Control (*n* = 17)	Pyramid	4.23 (±0.8) **b**	
2018	*S. riobrave* (*n* = 19)	Pyramid	1.05 (±0.5) **a**	Wilcoxon Matched Pairs Test; z = 3.33, *p* < 0.001.
	Control (*n* = 16)	Pyramid	6.38 (±1.3) **b**	
**2019**	*S. riobrave* (*n* = 5)	Pyramid	1.00 (±0.4) **a**	Kruskal-Wallis H = 10.19; *p* = 0.017. Kruskal-Wallis
	*S. carpocapsae* (*n* = 5)	Pyramid	2.60 (±1.6) **a**	
	*S. feltiae* (*n* = 5)	Pyramid	6.40 (±0.7) **b**	
	Control (*n* = 5)	Pyramid	6.20 (± 0.7) **b**	

## Appendix A

**Characterization of soil types (study 2)**: Soils monitored in this study were mapped by the USDA-NRCS, and the mapping data for each farm was confirmed in the field. Soil information was provided by the USDA Official Series Descriptions (USDA-NRCS, 2020). Soils at Apple Hill farm predominantly consist of the Canterbury series (Oxyaquic Dystrudepts). Soils were well drained loams in general, and underlain by dense, loamy till. Soils at the Clark Brothers farm predominantly consist of the Shelburne series (Oxyaquic Dystrudepts). Soils were very deep, well drained, and formed in a loamy glacial till. Soils at Clarkdale farm predominantly consist of very deep, excessively drained soils formed in glaciofluvial materials. Soils at Gould farm predominantly consist of well drained loamy soils, which are underlain by dense, loamy till. Soils at Poverty Lane farm were evenly split between the Bernardston series (Typic Dystrochrepts) and Pittstown series (Aquic Dystrochrepts). The Bernardston series consists of very deep, well drained soils formed in till, derived mainly from dark gray phyllite, slate, or schist. The Pittstown series consists of moderately well drained soils formed in lodgement till derived mainly from slate, phyllite, shale, and schist. Soils at Scott farm consist mostly of the Marlow series (Oxyaquic Haplorthods), with some areas of the Tunbridge (Typic Haplorthods) series. The Marlow series consists of well drained soils that formed in loamy lodgment till on hills and mountains in glaciated uplands. The Tunbridge series consists of moderately deep, well drained soils on glaciated uplands. Soils at Cold Spring farm consist of areas of the three soil series: Scituate (Typic Dystrochrepts), Ridgebury (Aeric Endoaquepts), and Montauk (Oxyaquic Dystrudepts). The Scituate series consists of moderately well drained soils, formed in a loamy eolian influenced mantle of till, underlain by sandy lodgement till. The Ridgebury series consists of very deep, somewhat poorly and poorly drained soils formed in lodgment till derived mainly from granite, gneiss and/or schist. The Montauk series consists of well drained soils formed in lodgment or flow till, derived primarily from granitic materials with lesser amounts of gneiss and schist.

## References

[1] Racette G., Chouinard G., Vincent C., Hill S.B. (1992). Ecology and management of plum curculio in apple orchards. Phytoprotection.

[2] Leskey T.C., Chouinard G., Vincent C., Aluja M., Leskey T.C., Vincent C. (2009). Monitoring and management of the apple maggot fly and the plum curculio: Honouring the legacy of R.J. Prokopy. Biorational Tree-Fruit Pest Management.

[3] Vincent C., Chouinard G., Hill S.B. (1999). Progress in plum curculio management: A review. Agric. Ecosyst. Environ..

[4] Prokopy R.J., Mason J.L., Christie M., Wright S.E. (1996). Arthropod pest and natural enemy abundance under second-level versus first-level integrated pest management practices in apple orchards: A 4-year study. Agric. Ecosyst. Environ..

[5] Reissig W.H., Nyrop J.P., Straub R. (1998). Oviposition model for timing insecticide sprays against plum curculio in New York State. Environ. Entomol..

[6] Piñero J.C., Agnello A.M., Tuttle A., Leskey T.C., Faubert H., Koehler G., Los L., Morin G., Leahy K., Cooley D.R. (2011). Effectiveness of odor-baited trap trees for plum curculio (Coleoptera: Curculionidae) monitoring in commercial apple orchards in the Northeast. J. Econ. Entomol..

[7] Leskey T.C., Wright S.E., Saguez J., Vincent C. (2013). Impact of insecticide and fungicide residue contact on plum curculio, *Conotrachelus nenuphar* (Herbst), mobility and mortality: Implications for pest management. Pest Manag. Sci..

[8] Leskey T.C., Piñero J.C., Prokopy R.J. (2008). Odor-baited trap trees: A novel management tool for the plum curculio, *Conotrachelus nenuphar* (Herbst) (Coleoptera: Curculionidae). J. Econ. Entomol..

[9] Shapiro-Ilan D.I., Mizell R.F., Campbell J.F. (2002). Susceptibility of the plum curculio, *Conotrachelus nenuphar*, to entomopathogenic nematodes. J. Nematol..

[10] Shapiro-Ilan D.I., Mizell R.F., Cottrell T.E., Horton D.L. (2004). Measuring field efficacy of *Steinernema feltiae* and *Steinernema riobrave* for suppression of plum curculio, *Conotrachelus nenuphar*, larvae. Biol. Control.

[11] Shapiro-Ilan D.I., Leskey T.C., Wright S.E. (2011). Virulence of entomopathogenic nematodes to plum curculio, *Conotrachelus nenuphar*: Effects of strain, temperature, and soil type. J. Nematol..

[12] Shapiro-Ilan D.I., Wright S.E., Tuttle A.F., Cooley D.R., Leskey T.C. (2013). Using entomopathogenic nematodes for biological control of plum curculio, *Conotrachelus nenuphar*: Effects of irrigation and species in apple orchards. Biol. Control.

[13] Prokopy R.J., Chandler B.W., Dynok S.A., Piñero J.C. (2003). Odor-baited trap trees: A new approach to monitoring plum curculio (Coleoptera: Curculionidae). J. Econ. Entomol..

[14] Prokopy R.J., Jácome I., Gray E., Trujillo G., Ricci M., Piñero J.C. (2004). Using odor-baited trap trees as sentinels to monitor plum curculio (Coleoptera: Curculionidae) in apple orchards. J. Econ. Entomol..

[15] Piñero J.C., Wright S.E., Prokopy R.J. (2001). Response of plum curculio (Coleoptera: Curculionidae) to odor-baited traps near woods. J. Econ. Entomol..

[16] Piñero J.C., Prokopy R.J. (2003). Field evaluation of plant odor and pheromonal combinations for attracting plum curculios. J. Chem. Ecol..

[17] Eller F.J., Bartelt R.J. (1996). Grandisoic acid, a male-produced aggregation pheromone from the plum curculio, *Conotrachelus nenuphar*. J. Nat. Prod..

[18] Shapiro-Ilan D.I., Hazir S., Glazer I., Lacey L.A. (2017). Basic and applied research: Entomopathogenic nematodes. Microbial Agents for Control of Insect Pests: From Discovery to Commercial Development and Use.

[19] Vincent C., Chouinard C., Bostanian N.J., Morin Y. (1997). Peripheral zone treatments for plum curculio management: Validation in commercial apple orchards. Entomol. Exp. Appl..

[20] Prokopy R.J., Harp M., Hamilton A., Chandler B., Jacome I. (2003). Comparison of Avaunt versus Guthion in every-row versus perimeter-row sprays against key apple insect pests: 2002 results and project summary. Fruit Notes.

[21] Leskey T.C., Zhang A. (2007). Impact of temperature on plum curculio, *Conotrachelus nenuphar* Herbst (Coleoptera: Curculionidae) responses to odor-baited traps. J. Econ. Entomol..

[22] Piñero J., Bigurra E., Jácome I., Trujillo G., Prokopy R.J. (2004). Are adult plum curculios capable of overwintering within apple orchards?. Fruit Notes.

[23] Decagon Inc. Decagon’s 5TE Water Content, Temperature, and Electrical Conductivity (EC) Sensor. http://manuals.decagon.com/Retired%20and%20Discontinued/Manuals/13509_5TE_Web.pdf.

[24] USDA-NRCS Official Series Descriptions. https://soilseries.sc.egov.usda.gov.

[25] Piñero J.C., Prokopy R.J. (2006). Temporal dynamics of plum curculio, *Conotrachelus nenuphar* (Herbst) (Coleoptera: Curculionidae) immigration into an apple orchard in Massachusetts. Environ. Entomol..

[26] Chouinard G., Hill S.B., Vincent C., Barthakur N.N. (1992). Border-row sprays against the plum curculio (Coleoptera: Curculionidae) in apple orchards: A behavioral study. J. Econ. Entomol..

[27] Leskey T.C., Hock V., Chouinard G., Cormier D., Leahy K., Cooley D., Tuttle A., Eaton A., Zhang A. (2014). Evaluating electrophysiological and behavioral responses to volatiles for improvement of odor-baited trap tree management of *Conotrachelus nenuphar* (Coleoptera: Curculionidae). Environ. Entomol..

[28] Rodriguez-Saona C., Nielsen A., Shapiro-Ilan D., Tewari S., Kyryczenko-Roth V., Firbas N., Leskey T. (2019). Exploring an odor-baited “Trap Bush” approach to aggregate plum curculio (Coleoptera: Curculionidae) injury in blueberries. Insects.

